# A pan-cancer analysis of the prognostic and immunological roles of matrix metalloprotease-1 (MMP1) in human tumors

**DOI:** 10.3389/fonc.2022.1089550

**Published:** 2023-01-13

**Authors:** Shuai Mao, Anliang Xia, Xuewen Tao, Dingde Ye, Jiamu Qu, Meiling Sun, Haowei Wei, Guoqiang Li

**Affiliations:** ^1^ Department of Hepatobiliary Surgery, Affiliated Drum Tower Hospital, Medical School, Nanjing University, Nanjing, China; ^2^ Department of Hepatobiliary Surgery, Medicine School of Southeast University Nanjing Drum Tower Hospital, Nanjing, China; ^3^ Department of Hepatobiliary Surgery, The First Affiliated Hospital of Anhui Medical University, Hefei, China

**Keywords:** MMP1, pan-cancer, prognosis, immune infiltration, molecular biology experiments

## Abstract

**Objective:**

Cancer remains the leading killer of human health worldwide. It has been shown that matrix metalloproteinase-1(MMP1) is related to poor prognosis in cancers such as BRCA, CESC and COAD. However, systematic pan-cancer analysis about the prognostic and immunological roles of MMP1 has not been explored. Here, the purpose of this study was to investigate the prognostic and immunological roles of MMP1 in pan-cancer and confirm cancer-promoting effect in pancreatic cancer.

**Methods:**

In our study, bioinformatics were first used to analyze data from multiple databases. Then, several bioinformatics tools were utilized to investigate the role of MMP1 in 33 tumor types. Finally, molecular biology experiments were carried out to prove the cancer-promoting effect of MMP1 in pancreatic cancer.

**Results:**

MMP1 expression was higher in tumor tissues than in control tissues in most tumor types. High expression of MMP1 was associated with poor overall survival (OS) and disease-free survival (DFS) in some tumor types. Further analysis of MMP1 gene mutation data showed that MMP1 mutations significantly influenced the prognosis of STAD. In addition, MMP1 expression was closely related to cancer-associated fibroblast (CAFs) infiltration in a variety of cancers and played an important role on immune infiltration score, tumor mutational burden (TMB) and microsatellite instability (MSI). Gene Ontology enrichment analysis indicated that these 20 genes were mainly related to extracellular structure organization/extracellular matrix organization/extracellular matrix disassembly/collagen metabolic process in the enriched biological processes. Finally, molecular biology experiments confirmed the cancer-promoting effect of MMP1 in pancreatic cancer.

**Conclusions:**

Our pan-cancer analysis comprehensively proved that MMP1 expression is related with clinical prognosis and tumor immune infiltration, and MMP1 can become a prognostic and immunological biomarker.

## Introduction

Recently, the incidence of cancer has been increasing worldwide (1), and cancer, as the main cause of death, has become increasingly prominent. China has become a veritable cancer country (2). As a populous country in the world, cancer data from China are not optimistic, and China ranks first in the world in terms of both new numbers and deaths. Therefore, it is urgent to find tumor biomarkers and targets to conquer cancer. For any possible tumor-associated gene, it is important to explore its clinical prognostic relevance and molecular pathogenic mechanisms, let alone its pan-cancer expression. The TCGA (The Cancer Genome Atlas) and GEO (Gene Expression Omnibus) databases provide functional genomics information for different cancers (**Table 1**), which allows us to carry out pan-cancer analysis on any gene of interest (3–5).

**Table 1:** Cancer abbreviations and the corresponding full name of abbreviations.

ACC	Adrenocortical carcinoma
BLCA	Bladder Urothelial Carcinoma
BRCA	Breast invasive carcinoma
CESC	Cervical squamous cell carcinoma and endocervical adenocarcinoma
CHOL	Cholangiocarcinoma
COAD	Colon adenocarcinoma
DLBC	Lymphoid Neoplasm Diffuse Large B-cell Lymphoma
ESCA	Esophageal carcinoma
GBM	Glioblastoma multiforme
HNSC	Head and Neck squamous cell carcinoma
KICH	Kidney Chromophobe
KIRC	Kidney renal clear cell carcinoma
KIRP	Kidney renal papillary cell carcinoma
LAML	Acute Myeloid Leukemia
LGG	Brain Lower Grade Glioma
LIHC	Liver hepatocellular carcinoma
LUAD	Lung adenocarcinoma
LUSC	Lung squamous cell carcinoma
MESO	Mesothelioma
OV	Ovarian serous cystadenocarcinoma
PAAD	Pancreatic adenocarcinoma
PCPG	Pheochromocytoma and Paraganglioma
PRAD	Prostate adenocarcinoma
READ	Rectum adenocarcinoma
SARC	Sarcoma
STAD	Stomach adenocarcinoma
SKCM	Skin Cutaneous Melanoma
TGCT	Testicular Germ Cell Tumors
THCA	Thyroid carcinoma
THYM	Thymoma
UCEC	Uterine Corpus Endometrial Carcinoma
UCS	Uterine Carcinosarcoma
UVM	Uveal Melanoma

Matrix metalloproteinase-1 (MMP1) is a Zn^2+^-dependent endopeptidase that is secreted into the extracellular matrix and specifically degrades type I, II, and III collagen fibers (6). In malignant tumors, proliferation of tumor cells is usually accompanied by high expression of MMP1 and MMP1 is activated to degrade the extracellular matrix and attenuate hindrance during cell movement, which in turn promotes tumor invasion and metastasis (7, 8). Although stromal cells in the tumor microenvironment, such as cancer-associated fibroblasts, also degrade and remodel the stroma, these stromal cells also need to be activated through signal transduction pathways. MMP1 can modulate these cellular behaviors by activating receptor signal transduction pathways (9). Lu et al. found that MMP1 coordinates paracrine signaling cascades to regulate the bone microenvironment and facilitate osteoclastogenesis and bone metastasis (10). Juncker-Jensen et al. showed that a tumor MMP-1/endothelial PAR1 axis promotes intravasation and vascular dissemination (11). MMP1 is also involved in G protein signal transduction, regulating tumor angiogenesis, and promoting tumor cell resistance (12, 13). Mechanistic studies of MMP1 in several tumors, including BRCA, CESC and COAD, have already been reported (10, 14, 15).

However, systematic pan-cancer analysis based on big data on the correlation between MMP1 expression and clinical manifestations of various tumors has not been explored. Here, we analyzed the correlation between MMP1 and clinical manifestations of different cancers from the aspects of differential gene expression, survival prognosis of cancer patients, gene mutation and tumorigenesis correlation, immune infiltration correlation, and gene-related cellular pathways. Also, we explored its potential mechanism of action in tumors and its possibility as a clinical molecular indicator of tumors (16). At last, we proved the MMP1 expression and cancer-promoting function in pancreatic cancer using biological experiments. Therefore, this study reveals the MMP1 expression and prognosis in human cancer, the relationship between MMP1 expression and tumor immunity, and the cancer-promoting effect of MMP1 in pancreatic cancer.

## Materials and methods

### MMP1 mRNA expression analysis

We utilized Tumor Immune Estimation Resource version 2 (TIMER2) to analyze MMP1 expression levels between tumors and paired normal tissues. In tumor types with no or limited normal tissues, we investigated MMP1 expression by using ‘Gene Expression Profile’ section of Gene Expression Profiling Interactive Analysis (version 2) (GEPIA2) to combine data from normal tissues in the Genotype-Tissue Expression (GTEx) database, under parameters of log_2_FC (fold change) cutoff = 1 and P-value cutoff = 0.01. We also used ‘Pathological Stage Plot’ section to obtain MMP1 expression in TCGA cancers at each pathological stage.

### MMP1 protein expression analysis and immunohistochemistry staining

UALCAN tool was utilized to perform protein expression analysis. Protein expression data were from Clinical Proteomic Tumor Analysis Consortium (CPTAC) dataset. Besides, we downloaded and analyzed immunohistochemical images of MMP1 protein expression in human pancreatic normal tissues and tumor tissues from the Human Protein Atlas (HPA).

### Survival prognosis analysis and ROC curve analysis

We obtained overall survival (OS) and disease-free survival (DFS) significance maps and Kaplan–Meier (K-M) survival plots of MMP1 by utilized ‘Survival Analysis’ section of GEPIA2. We used cut-off value of 50% to split the high and low expression cohorts. Log-rank test was used to perform hypothesis testing.

We obtained RNA-seq data in transcripts per million reads (TPM) format from TCGA and GTEx uniformly processed by the Toil process (17) in UCSC XENA. Tumor data from TCGA and corresponding normal tissue data from GTEx were extracted. Expression comparisons between samples were performed after log2 transformation of RNA-seq data in TPM format. Finally, we utilized R package pROC (version 1.17.0.1) to analyze data and R package ggplot2 (version: 3.3.3) to visualize the results.

### Genetic alteration analysis

The cBioPortal tool was employed to analyze MMP1 genetic alterations. Alteration frequency and type and mutated site for MMP1 from TCGA Pan-Cancer Atlas Studies were obtained and analyzed. We input ‘MMP1’ to ‘Quick Search’ module, and genetic alterations and mutated site data can be obtained from ‘Cancer Types Summary’ and ‘Mutations’ modules.

According to whether MMP1 was mutated or not, we divided the limited tumor sample data available from the TCGA into MMP1 altered and unaltered groups and compared their prognoses, including OS, disease-specific survival (DSS) and progress-free survival (PFS).

### Immune infiltration analysis

MCPCOUNTER, TIDE and EPIC algorithms were employed to investigate the correlation between MMP1 expression and infiltration of CAFs in the ‘Immune Association’ section of TIMER2.

We obtained the tumor dataset from the UCSC database. Immediately, the MMP1 expression data in each sample were extracted. Then, we chose samples from which the samples were derived from Primary Blood Derived Cancer-Peripheral Blood, Primary Tumor Correlation test and Metastatic samples of TCGA-SKCM. Besides, tumor gene expression profiles were extracted. Subsequently, we mapped them to GeneSymbol. Finally, R package ESTIMATE (version 1.0.13) was used to calculate stromal, immune, and estimate scores, and the corr. test function of R package psych (version 2.1.6) was utilized to calculate Pearson’s correlation between MMP1 expression and immune infiltration scores.

### Correlation analysis of MMP1 expression with TMB and MSI

We obtained tumor data from UCSC, and MMP1 expression data were extracted. Then we chose samples from which the samples were derived from Primary Blood Derived Cancer-Peripheral Blood and Primary Tumor. We also obtained the Simple Nucleotide Variation dataset of level4 for TCGA samples from GDC treated by MuTect2 software. TMB score were calculated by using maftools R package (version 2.8.05). Besides, we obtained MSI score from a previous study (18). Finally, we explored Spearman’s correlations between TMB and MSI and MMP1 expression.

### Protein network and MMP1-related gene enrichment analysis

GeneMANIA website was utilized to find functionally similar genes using a large body of genomics and proteomics data, and we used it to search for genes that are functionally similar to MMP1. Also, a human MMP1-binding protein co-expression network can be obtained by STRING tool. Next, we set the main parameters: max number of interactors to show (‘no more than 20 interactors’), minimum required interaction score [‘highest confidence (0.700)’], active interaction sources (select all), meaning of network edges(‘evidence’), and Network type (‘full STRING network’). All gene symbols were used as input gene symbols for Gene Ontology pathway enrichment analysis with R package clusterProfiler (version: 3.14.3). In the end, we utilized R package ggplot2 (version: 3.3.3) to visualize the results.

### Cell culture and transfection

Human PAAD cell lines (AsPC-1, PANC-1 and SW1990) were purchased from Shanghai Institute of Biochemistry and Cell Biology. Cells were passaged and cryopreserved by the Central Laboratory of Hepatobiliary Pancreas, Nanjing Drum Tower Hospital. Human normal pancreatic cell line (HPDE) and PAAD cell lines (Capan-2 and BxPC-3) were derived from our laboratory. Cells were cultured in DMEM (Dulbecco’s modified Eagle’s medium) or RPMI 1640 containing 10% fetal bovine serum (FBS) and 1% penicillin–streptomycin (WISENT, China). All cells were cultured at 37°C with 5% CO2. Moreover, all siRNAs (si-NC, si-MMP1) that purchased from RiboBio (Guangzhou, China) were transfected into BxPC-3 and Capan-2 cells by using Lipofectamine 3000 (Invitrogen, USA). At 2 days after transfection, cells were collected for following experiments. The sequences of siRNAs were displayed in **Supplementary Table 1**.

### Western blotting analysis

RIPA buffer (Beyotime, China) was used to lyse cells to extract total protein. The treated protein samples were then subjected to SDS-PAGE. We transferred the separated protein onto PVDF membranes after electrophoresis. Membranes were blocked with blocking solution (5% skim milk) and then primary antibodies (MMP1, Abcam; GAPDH, Abcam) were used to incubate membranes overnight. After incubation with secondary antibodies, proteins were visualized with high-sensitivity electrochemiluminescence agent (Beyotime, China) followed by autoradiography.

### Wound healing assay

Culture-Inserts 2 Well for self-insertion (Ibidi, Germany) were placed into wells of 6-well plates. Cells were digested with trypsin, and resuspended with DMEM medium containing 1% FBS and 1% penicillin–streptomycin. 3×10^4^ cells were seeded in each well of the insert. After one night, the insert was removed. Cell migration was observed by photography in an Olympus CKX41 inverted microscope at 0 and 48 h. The scratch area at 0 and 48 h was measured using Image J software.

### Transwell assay

Transwell inserts (Corning, USA) were coated with Matrigel (BD biosciences, USA). 1 × 10^5^ cells were seeded in the upper chamber with serum-free DMEM, and serum containing DMEM was added to the lower chamber. Cells were incubated in 37°C incubator for 24h. Then, cells attached to the chamber were fixed in 4% paraformaldehyde. After 20 minutes, the chamber was moved into 0.1% crystal violet. Finally, cells in the upper chamber and Matrigel were gently removed with cotton swabs, and the total number of invaded cells was counted under an Olympus IX73 inverted microscope.

### CCK8 detection

PAAD cells transfected with si-MMP1 and si-NC in logarithmic growth phase were seed in 96-well plates at 1000 cells/well density, and 5 multiple wells were designed for each group. After incubation for 24h 48h and 72h, a volume of 10 μL CCK8 solution (Vazyme, China) was added to each well and incubated in an incubator for 1 h. The wavelength at OD450 was measured in a multiplate reader.

### Flow cytometry assay

According to manufacturer’s instructions, samples were stained with Annexin V-FITC and propidium iodide (Vazyme, China). Then, an Aria flow cytometer (BD Biosciences, USA) was utilized to display the apoptotic cell population. Finally, FlowJo software was used to analyze data.

### Statistical analysis

Data were analyzed using GraphPad Prism, version 6.00 and SPSS, version 20.0. We employed One-way ANOVA to compare multiple groups, and Student’s t-test to compare two groups. Data were reported as means ± SD. *P ≤ 0.05* was considered statistically significant.

## Results

### Expression analysis of MMP1

**Figure 1:**
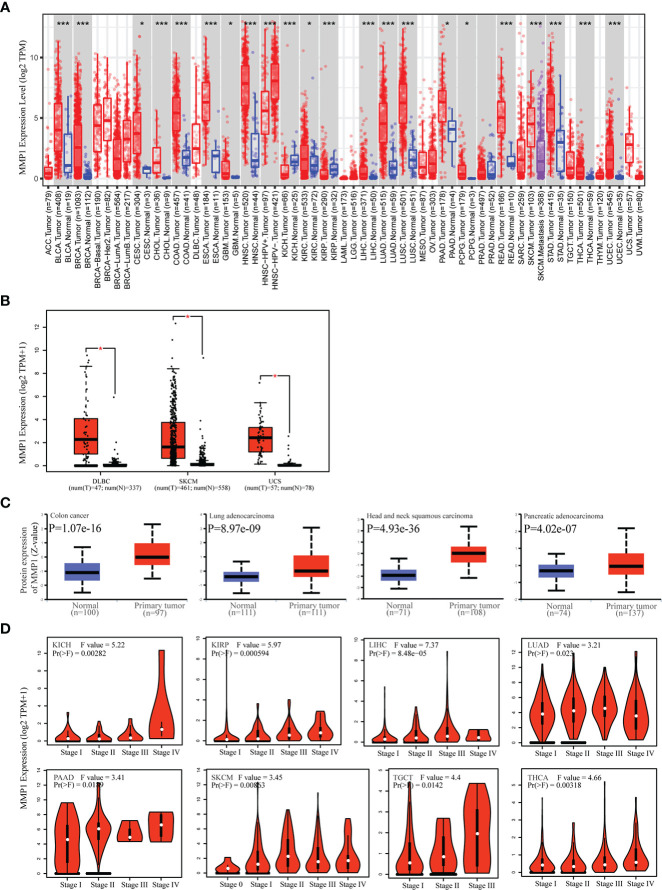
Differences in MMP1 mRNA expression in human tumor tissues and normal tissues. **(A)** Analysis and comparison of MMP1 mRNA expression in different tumor tissues and normal tissues in TCGA database. *P < 0.05, ***P < 0.001. **(B)** Boxplots of the differential MMP1 mRNA expression in 3 cancer types from the GTEx database and TCGA database. *P < 0.05. **(C)** Protein expression level of MMP1 in normal tissues and Colon cancer, LUAD, HNSC and PAAD. Protein data from CPTAC was extracted and analyzed using UALCAN tool. **(D)** Correlation between MMP1 expression and pathological tumor stages of KICH, KIRP, LIHC, LUAD, PAAD, SKCM, TGCT and THCA from TCGA datasets. Log2 (TPM + 1) was applied for log-scale.

TIMER2 was utilized to investigate MMP1 expression levels between tumor tissues and control tissues from the TCGA database. MMP1 expression levels were significantly higher in 19 tumor types than in corresponding controls. Notably, the mRNA levels of MMP1 were only lower in KICH than in control tissues. In addition, MMP1 expression levels were not different in PRAD compared to normal tissues (**Figure 1A**). As for tumor types without normal tissue data in TCGA, we analyzed MMP1 expression by combining TCGA and GTEx data. We found higher expression of MMP1 in tumor tissues from DLBC, SKCM and UCS (**Figure 1B**).

Next, we obtained MMP1 protein levels by utilizing the CPTAC dataset from the National Cancer Institute. The data revealed that MMP1 protein levels in COAD, HNSC, LUAD and PAAD were significantly higher than in control tissues (**Figure 1C**). Moreover, GEPIA2 were utilized to investigate the correlation between MMP1 expression and pathological stages. MMP1 expression was significantly related to the tumor stage in 9 cancer types (**Figure 1D**). Taken together, these data showed that MMP1 expression was elevated in most tumor types from TCGA.

### Diagnostic and prognostic roles of MMP1 in cancer patients

**Figure 2:**
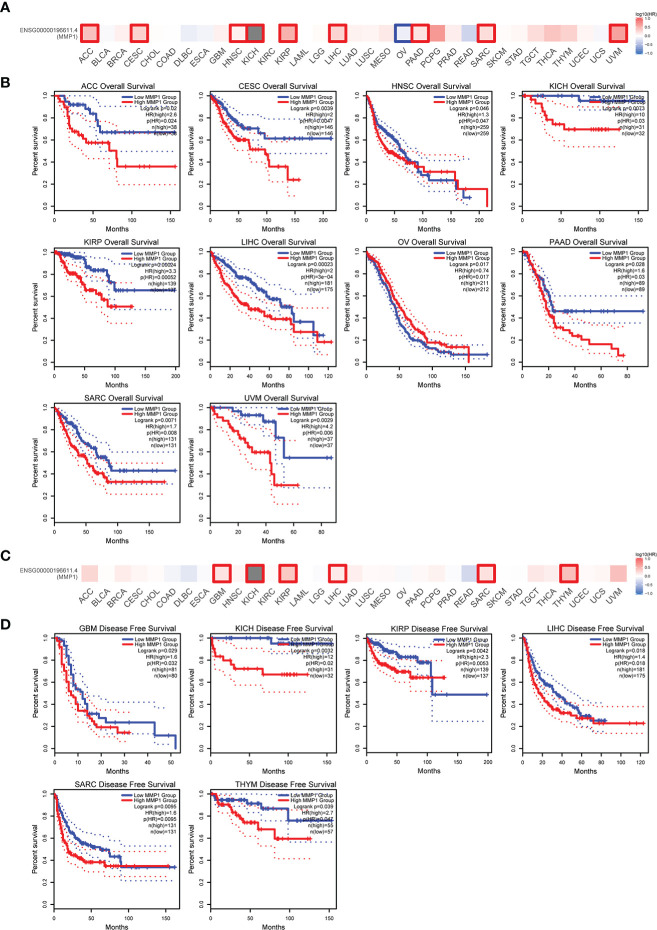
Correlation between MMP1 expression level and patient overall survival (OS) and disease-free survival (DFS) in TCGA tumors. The positive results of overall survival map **(A)** and OS curves **(B)** were listed. The positive results of disease-free survival map **(C)** and DFS curves **(D)** were also displayed.

To understand the correlation between MMP1 expression and prognosis, we investigated the correlation between MMP1 high and low expression groups and prognosis using the TCGA dataset. High MMP1 expression was related to poor OS in 9 tumor types. However, in OV, high MMP1 expression may be related to better OS (**Figures 2A, B**). In addition, high MMP1 expression was related to poor DFS in 6 tumor types (**Figures 2C, D**).

**Figure 3:**
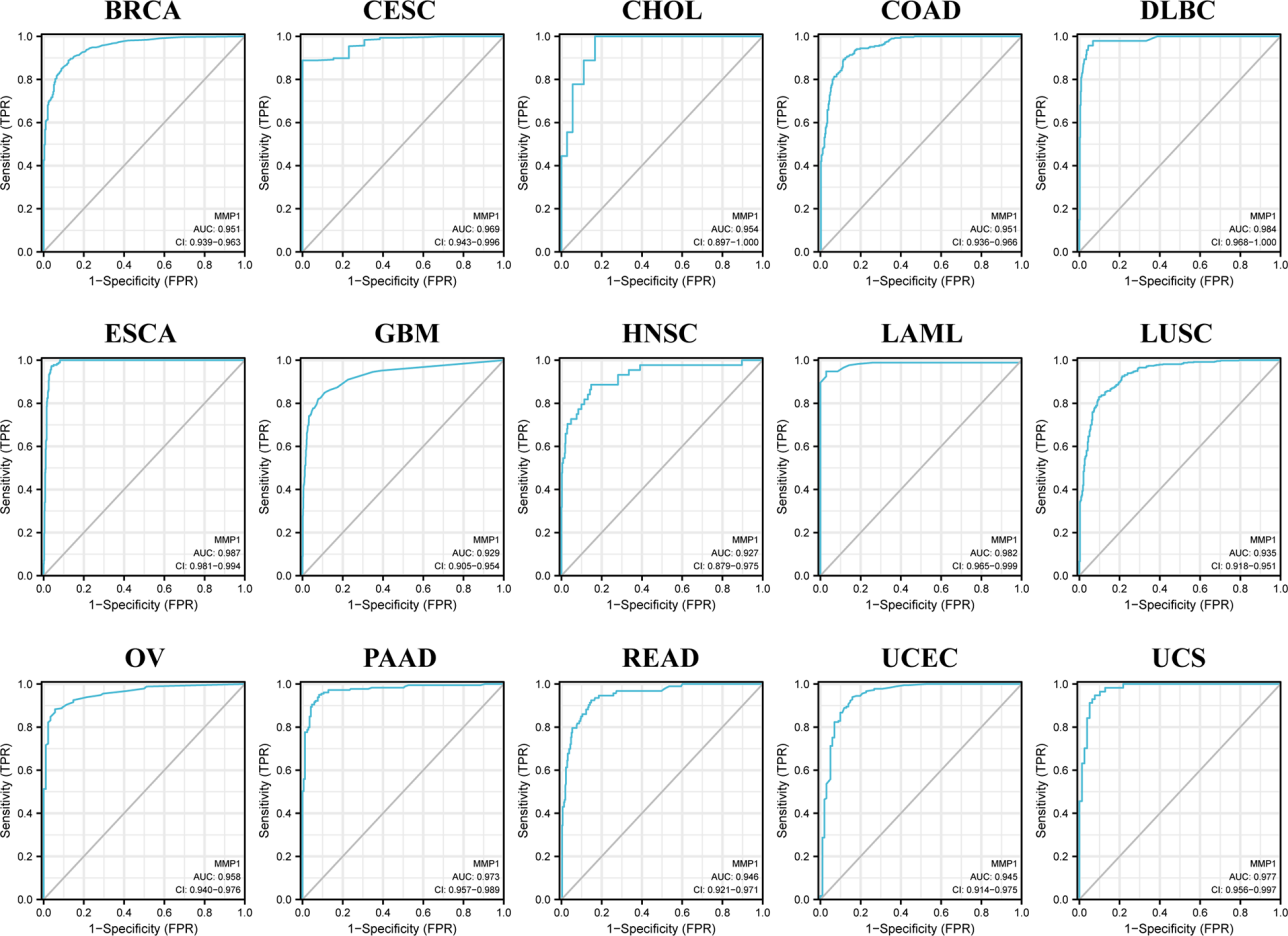
Receiver Operating Characteristics (ROC) analysis of MMP1 genes in TCGA database.

We also used ROC curves to evaluate the diagnostic accuracy of MMP1 in human tumors. The data suggested that MMP1 had a high accuracy (AUC > 0.9) in predicting the diagnosis in 15 cancer types (**Figure 3**).

### The genetic alteration of MMP1 in TCGA tumor types

**Figure 4:**
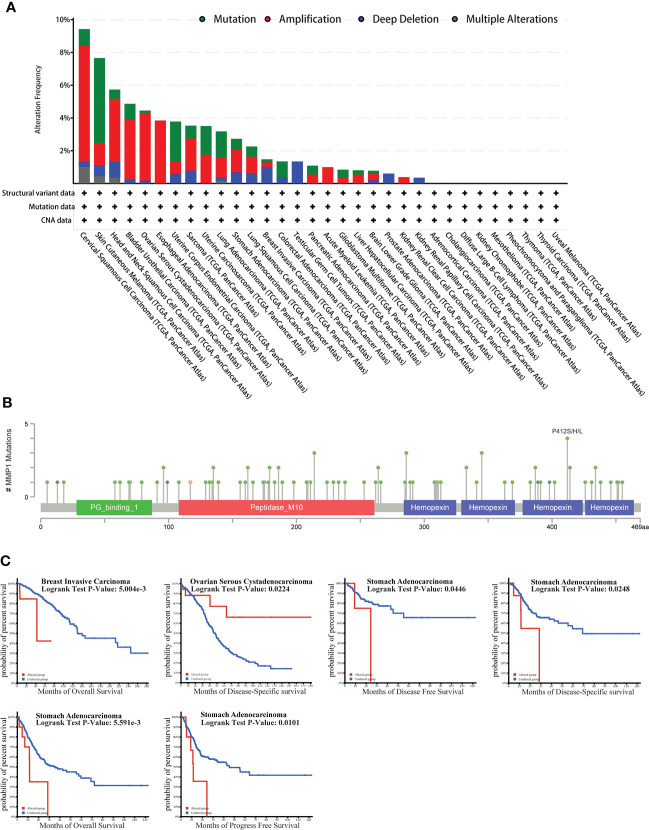
Correlation between MMP1 and mutation in TCGA tumors based on the cBioPortal database. **(A)** The alteration frequency with mutation type in TCGA pan-cancer datasets. **(B)** The mutation types, number, and sites of the MMP1 genetic alterations were displayed. **(C)** Analysis of the correlation between mutation status and OS (Overall survival), DSS (Disease-specific survival), DFS (Disease-free survival) and PFS (Progression-free survival) of 3 cancer types using the cBioPortal tool, and the positive results of Kaplan-Meier curves were listed.

As we know, genetic alterations promote tumor development. To investigate the genetic alterations of MMP1, we utilized the cBioPortal website to analyze data from TCGA. We found that MMP1 gene alterations were most frequent (> 8%) in cervical squamous cell carcinoma among all tumors, and the predominant type of alterations was amplification (**Figure 4A**). As shown in **Figure 4B**, the most frequently mutated region (P412S/H/L) in MMP1 protein was mutated four times in total.

Moreover, we further investigated the relationship between MMP1 gene alterations and prognosis, and we presented meaningful results in **Figure 4C**. STAD patients with MMP1 gene alterations had poor DFS, DSS, OS, and PFS. In patients with BRCA, people with MMP1 gene alterations had a poor OS. However, with MMP1 gene alterations, OV patients had a better prognosis in DSS.

### Immune infiltration analysis

**Figure 5:**
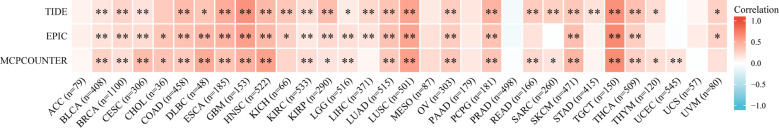
Correlation between MMP1 expression and CAFs immune infiltration. TIDE, EPIC and MCPCOUNTER algorithms was used to calculate the correlation between MMP1 expression and CAFs immune infiltration in all tumor types from TCGA. *P < 0.05, **P < 0.01.

Malignant solid tumor tissue includes many types of cells. In addition to tumor cells, stromal cells and infiltrating immune cells are also considered to play an important role in tumor progression. It is unclear whether the MMP1 expression influences the recruitment of immune cells. We used TIDE, EPIC and MCPCOUNTER algorithms to analyze the correlation between immune cells infiltration and MMP1 expression in pan-cancer based on the TIMER2. We found that CAFs was positively related to MMP1 expression in 31 tumor types, and CAFs was significantly related to MMP1 expression in 18 tumor types (**Figure 5**).

We also analyzed immune cells and stromal cells to determine the stromal, immune, and estimate scores. Eventually, we found a significant correlation between MMP1 expression and stromal score in 24 tumor types, and all of them were positively correlated (**Supplementary Figure 1**). Moreover, MMP1 expression was positively and significantly related to immune score in 16 tumor types. However, THYM and TGCT were negatively related (**Supplementary Figure 2**). In the relationship between MMP1 expression and estimate score, MMP1 expression was significantly associated with estimate score in 22 tumor types. A negative correlation was found in THYM, and other tumor types were positively related to MMP1 expression (**Supplementary Figure 3**). Above all, MMP1 expression had a correlation with immune infiltration.

### Relationship between MMP1 expression and TMB and MSI

**Figure 6:**
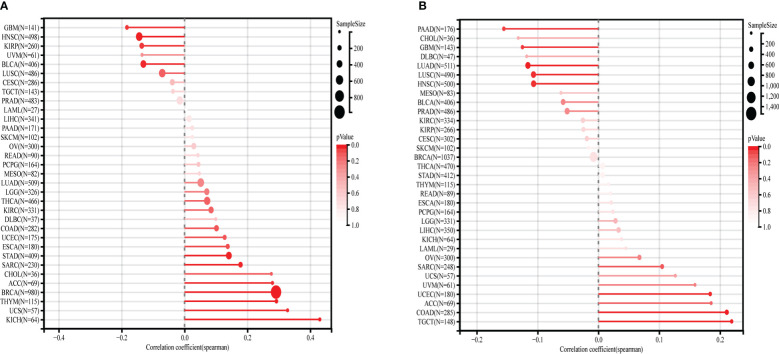
Correlation between MMP1 expression and TMB **(A)** and MSI **(B)**.

We calculated the spearman correlation between MMP1 expression and TMB and MSI, and observed a positive correlation between MMP1 expression and TMB in ACC (r=0.30, p=2.0e-2), BRCA (r=0.29, p=1.35e-20), KICH (r=0.43, p=3.9e-4), SARC (r=0.18, p=6.8e-3), STAD (r=0.14, p=4.3e-3), THYM (r=0.29, p=1.5e-3) and UCS (r=0.33, p=1.3e-2). In contrast, MMP1 expression was significantly and negatively related to TMB in BLCA (r=-0.13, p=8.4e-3), GBM (r=-0.18, p=3.0e-2), HNSC (r=-0.14, p=1.3e-3) and KIRP (r=-0.14, p=2.8e-2) (**Figure 6A**). In COAD (r=0.21, p=3.6e-7), TGCT (r=0.22, p=7.4e-3) and UCEC (r=0.18, p=1.3e-2), MMP1 expression had a positive correlation with MSI, whereas in GBM (r=-0.12, p=3.5e-2), HNSC (r=-0.11, p=1.7e-2), LUAD (r=-0.12, p=2.0e-4), LUSC (r=-0.11, p=1.8e-2) and PAAD (r=-0.16, p=3.9e-2), it was adversely connected with MSI (**Figure 6B**).

### Enrichment analysis of genes related to MMP1

**Figure 7:**
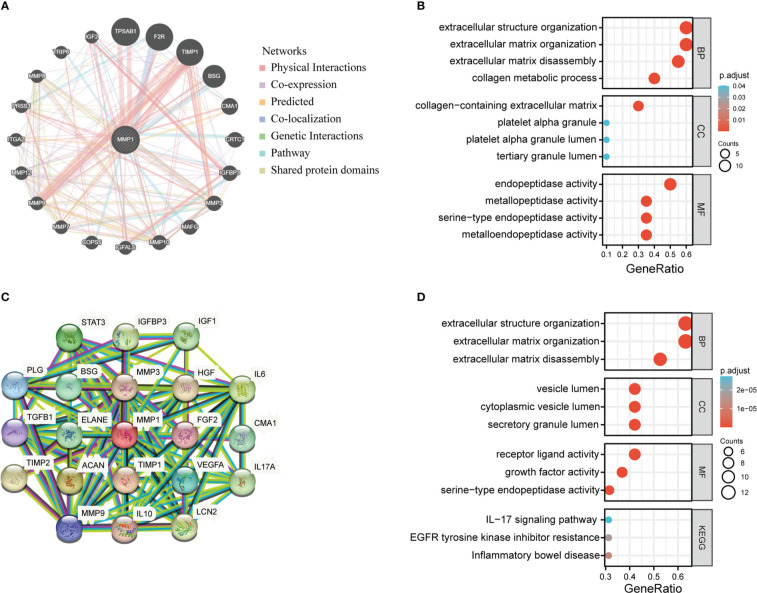
MMP1-related partners enrichment analysis. **(A)** The potential interaction molecular network of MMP1 was created using the GeneMANIA. **(B)** GO|KEGG pathway analysis of the molecules interacted with MMP1. **(C)** STRING protein network map of MMP1-binding proteins. **(D)** GO/KEGG pathway analysis of MMP1 and MMP1-related partners.

We extracted the top 20 genes most closely related to MMP1 *via* GeneMANIA (**Figure 7A**). Subsequently, GO enrichment analysis showed that these 20 genes were mainly correlated with extracellular structure organization/extracellular matrix organization/extracellular matrix disassembly/collagen metabolic process in the enriched biological processes (BP), and the enriched cellular components (CC) were mainly associated with collagen−containing extracellular matrix, platelet alpha granule platelet alpha granule lumen and tertiary granule lumen. Besides, molecular functions (MF) were related to endopeptidase activity, metallopeptidase activity, serine−type endopeptidase activity and metalloendopeptidase activity (**Figure 7B**).

Moreover, we utilized the STRING tool to obtain 20 MMP1-interacting proteins and create a PPI network, which was showed in **Figure 7C**. These proteins were also enriched in extracellular structure organization/extracellular matrix organization/extracellular matrix disassembly, and the major enriched KEGG pathways were related to IL−17 signaling pathway, EGFR tyrosine kinase inhibitor resistance, and inflammatory bowel disease (**Figure 7D**).

### The expression of MMP1 in PAAD cells and effect of MMP1 on migration, invasion, proliferation and apoptosis of PAAD cells

**Figure 8:**
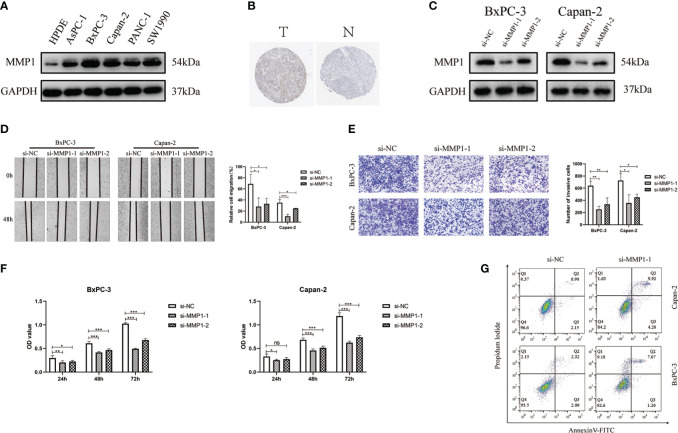
MMP1 is up-regulated in PAAD cells and is associated with proliferation, migration and invasion of PAAD cells. **(A)** The results of Western blotting confirmed the expression of MMP1 was higher in PAAD cells. **(B)** Immunohistochemistry image of MMP1 in normal pancreatic tissues and PAAD tissues. **(C)** Western blotting results showed the efficiency of siRNAs. **(D)** The results of wound-healing assays indicated the siMMP1 could restrain the migration of Capan-2 and BxPC-3 cells. **(E)** The results of transwell assays showed the ability of invasion in Capan-2 and BxPC-3 cells was decreased after transfection with siMMP1. **(F)** The proliferation of BxPC-3 and Capan-2 cells was detected by CCK8 assays **(G)** Flow cytometry results showed that MMP1-siRNA promoted PAAD cell apoptosis. Data were presented as mean ± SD. ns, non-significant, *P < 0.05, **P < 0.01 ***P < 0.001.

To validate the function of MMP1, we chose human pancreatic cancer for further investigation. Compared with HPDE, WB results suggested that MMP1 protein had a high expression in pancreatic cancer cells (**Figure 8A**). We also obtained the immunohistochemical pictures of MMP1 expression levels in pancreatic cancer from the HPA database (**Figure 8B**). Immunohistochemical results showed that MMP1 was overexpressed in pancreatic cancer. Then, we designed an siRNA against MMP1 and transfected it into Bxpc-3 and Capan-2 cells to examine the effect of MMP1 in PAAD cells. The results of WB demonstrated that siRNA had efficiency in inhibiting MMP1 expression (**Figure 8C**). Next, we found that silence of MMP1 could reduce the PAAD cells migration and invasion ability (**Figures 8D, E**). Subsequently, we examined the changes in cell viability after transfection in the normal cell group and the siMMP1-transfected group using the CCK8 kit. The results showed that the proliferation ability of PAAD cells was significantly reduced after 48 hours of MMP1 knockdown (**Figure 8F**). Meanwhile, we used flow cytometry to demonstrate that the knockdown of MMP1 also promoted PAAD cells apoptosis (**Figure 8G**). Therefore, these results indicated that MMP1 promoted the metastasis and inhibited the apoptosis of PAAD cells.

## Discussion

In our study, we utilized different databases such as TCGA, GEO and HPA to comprehensively investigate the molecular characteristics of MMP1 in human tumors for the sake of investigating the value of MMP1 in clinic prognosis and immunotherapy.

Our findings revealed that MMP1 expression was higher in 22 tumor types and only lower in KICH than in paired normal tissues. In addition, UALCAN database was used to confirm that protein levels of MMP1 were higher in COAD, HNSC, PAAD, and LUAD tumor tissues than in corresponding controls. Moreover, high MMP1 expression was associated with advanced clinical stages in 8 tumor types. Several studies have proved that MMP1 may be related to poor prognosis in tumors such as BRCA, CESC and COAD (10, 14, 15). In our study, we performed KM survival analysis using multiple databases and found that in general, high MMP1 expression predicted poor OS and DFS outcomes. Only in OV, high MMP1 expression expressed a better OS outcome. However, A previous study came to the opposite conclusion (19). In this study published in 2017, the investigators found that high MMP1 mRNA expression was significantly associated with poor OS in OV using KMplot software. Recently, we used KMplot software to perform the analysis again, but did not draw conclusions consistent with this study, which is mainly due to the continuous update of database. So, this more reflects the importance of biological experiments for the verification of bioinformatics analysis. In addition, we employed ROC curves to explore the predictive accuracy of MMP1 in tumors. The results demonstrated that MMP1 had high predictive accuracy (AUC > 0.9) in 15 tumor types. Combined with survival analysis and ROC curve analysis, we concluded that MMP1 expression was related to the prognosis of OS or DFS in CESC, GBM, HNSC, OV, and PAAD, and had high diagnostic value. In LIHC, MMP1 expression was associated with OS and DFS outcomes, which is consistent with a previous study (20). However, ROC curves showed that MMP1 didn’t have a high predictive accuracy in LIHC, and thus the prognostic role of MMP1 in HCC remained of concern. Interestingly, we found that MMP1 expression was low in KICH, which is consistent with a prior study (21), but high expression of MMP1 was related to advanced clinical stage, and had poor prognosis. Therefore, we speculated that MMP1 may be involved in regulating a negative feedback regulatory mechanism in KICH. We also found no statistical differences in the protein levels of MMP1 from most tumor types, which should be related to the fact that protein expression is regulated at multiple levels.

It has been shown that genetic alterations have an important impact on the cancer prognosis (22, 23). This research also showed that MMP1 had the highest mutation frequency in CESC, and the mutation frequency in HNSC was third in all tumor types, which was agreed with earlier studies reporting that genetic alterations in MMP1 were associated with HNSC (24). We further analyzed the effect of MMP1 mutation on the prognosis and survival of cancer patients. Although MMP1 expression had no effect on the prognosis of STAD, we found that MMP1 mutation had an effect on OS, DFS, DSS and PFS of STAD. No other researchers have yet investigated the role of MMP1 mutation in STAD.

Numerous studies have shown that the tumor microenvironment (TME) has an important influence on tumor development, in addition to being affected by the characteristics of tumor cells themselves. As an important component of TME, CAF also affects the development of tumors (25). On the one hand, CAFs can secrete a variety of cytokines to directly stimulate tumor cell proliferation and angiogenesis, and promote tumor growth, invasion and metastasis (26–28). On the other hand, they can remodel the extracellular matrix (ECM), form a permeability barrier of drugs, and prevent the effective penetration of anti-tumor drugs (29, 30). In our study, we used TIDE, EPIC and MCPCOUNTER algorithm to analyze the relationship between CAFs and MMP1 expression on the TIMER2 website. We found that CAFs infiltration positively correlated with MMP1 expression in 31 tumor types, and after three algorithms validation, CAFs was significantly related to MMP1 expression in 18 tumor types.

It has been reported that MMP1 could improve tumor resistance by regulating CAFs in HNSC (31). In our study, we also came to a consistent conclusion that CAFs are significantly positively correlated with MMP1 expression in HNSC. Another study showed that CAFs could regulate apoptotic signaling pathways in tumor cells to promote tumor growth. CAFs have been found to be involved in mediating the clinical resistance of various malignancies such as STAD (32), LUAD (33), LIHC (34) and COAD (35). These studies revealed the potential for MMP1 to become a clinical target. It has also been suggested that MMP1 participated in the regulation of tumor-associated macrophages in colon cancer (36), but our study did not find a close relationship between MMP1 and tumor-associated macrophages in pan-cancer. Also, we analyzed immune cells and stromal cells to obtain stromal, immune, and estimate scores in each tumor. In addition, we calculated spearman’s correlation between MMP1 expression and tumor mutational burden and microsatellite instability in each cancer type (37). The results suggested that MMP1 was associated with tumor immunity.

It has been reported that CAFs are important cellular components involved in ECM remodeling, which help tumor growth by increasing the deposition of certain components of ECM to induce stromal changes (38, 39). Through enrichment analysis of MMP1-related genes (40), we found that the main functions of MMP1 lay in extracellular structure/extracellular matrix organization/extracellular matrix disassembly/collagen metabolic process, which is consistent with prior studies. Combined with the phenomenon that MMP1 expression was closely associated with CAFs infiltration, we concluded that MMP1 not only acted to degrade extracellular matrix and promote tumor metastasis (41, 42), but also promoted the production of extratumoral matrix by CAFs, thereby promoting tumor development (43, 44).

In addition, we verified that MMP1 was highly expressed in PAAD cell lines using molecular biology experiments and was involved in migration, invasion, proliferation and apoptosis of PAAD cells. It was indicated that circular RNA circDLC1 could inhibit liver cancer development by interacting with MMP1 (45). Another study showed that MMP1 was involved in the cancer-promoting effect of TCONS_00012883 on colorectal cancer (46). These studies suggested that MMP1 may be able to be a prognostic and immune-related biomarker (47–49). However, although we explored the relationship of MMP1 expression with clinical outcome and tumor immune infiltration using several databases, this study still needs to go further to obtain more rigorous conclusions. We need more biological experiments to explore the specific mechanism of MMP1 in tumor progression (50). In addition, the specific effect of MMP1 on CAFs in different tumors needs further validations.

Overall, our study elucidated the correlation between MMP1 expression and clinical prognosis and immune infiltration. We also utilized biological experiments to validate the cancer-promoting effect of MMP1 in pancreatic cancer. It provides a theoretical basis for introducing MMP1 as a new prognostic and immunological biomarker.

## Conclusion

Our study comprehensively proved that MMP1 expression is related to clinical prognosis and tumor immune infiltration, and MMP1 can become a prognostic and immunological biomarker.

## Data availability statement

The original contributions presented in the study are included in the article/**Supplementary Material**. Further inquiries can be directed to the corresponding author.

## Author contributionss

SM, AX, XT and GL contributed to the conception of the study. SM, DY, MS, HW and JQ contributed materials and performed the experiment. SM and XT performed the data analyses. SM, AX and GL contributed significantly in writing the manuscript. All authors contributed to the article and approved the submitted version.
