# Correction: A pan-cancer analysis of the prognostic and immunological roles of matrix metalloprotease-1 (MMP1) in human tumors

**DOI:** 10.3389/fonc.2026.1887592

**Published:** 2026-07-20

**Authors:** Shuai Mao, Anliang Xia, Xuewen Tao, Dingde Ye, Jiamu Qu, Meiling Sun, Haowei Wei, Guoqiang Li

**Affiliations:** 1 Department of Hepatobiliary Surgery, Affiliated Drum Tower Hospital, Medical School, Nanjing University, Nanjing, China; 2 Department of Hepatobiliary Surgery, Medicine School of Southeast University Nanjing Drum Tower Hospital, Nanjing, China; 3 Department of Hepatobiliary Surgery, The First Affiliated Hospital of Anhui Medical University, Hefei, China

**Keywords:** MMP1, pan-cancer, prognosis, immune infiltration, molecular biology experiments

There was a mistake in **Figure 8** as published. Previously, we indeed downloaded the IHC images of MMP1 from the HPA database. However, when we searched again recently, these IHC images had disappeared, which is quite unusual. During the preparation of our manuscript, we were confident that the representative image we downloaded from the HPA database corresponded to MMP1. Now, **Figure 8B** in our article and **Figure 9A** in the Discover Oncology article appear to be identical, while they are used to represent IHC expression of different genes.The exact reason for this discrepancy remains unclear. The corrected **Figure 8** and its legend appear below.

**Figure 8:**
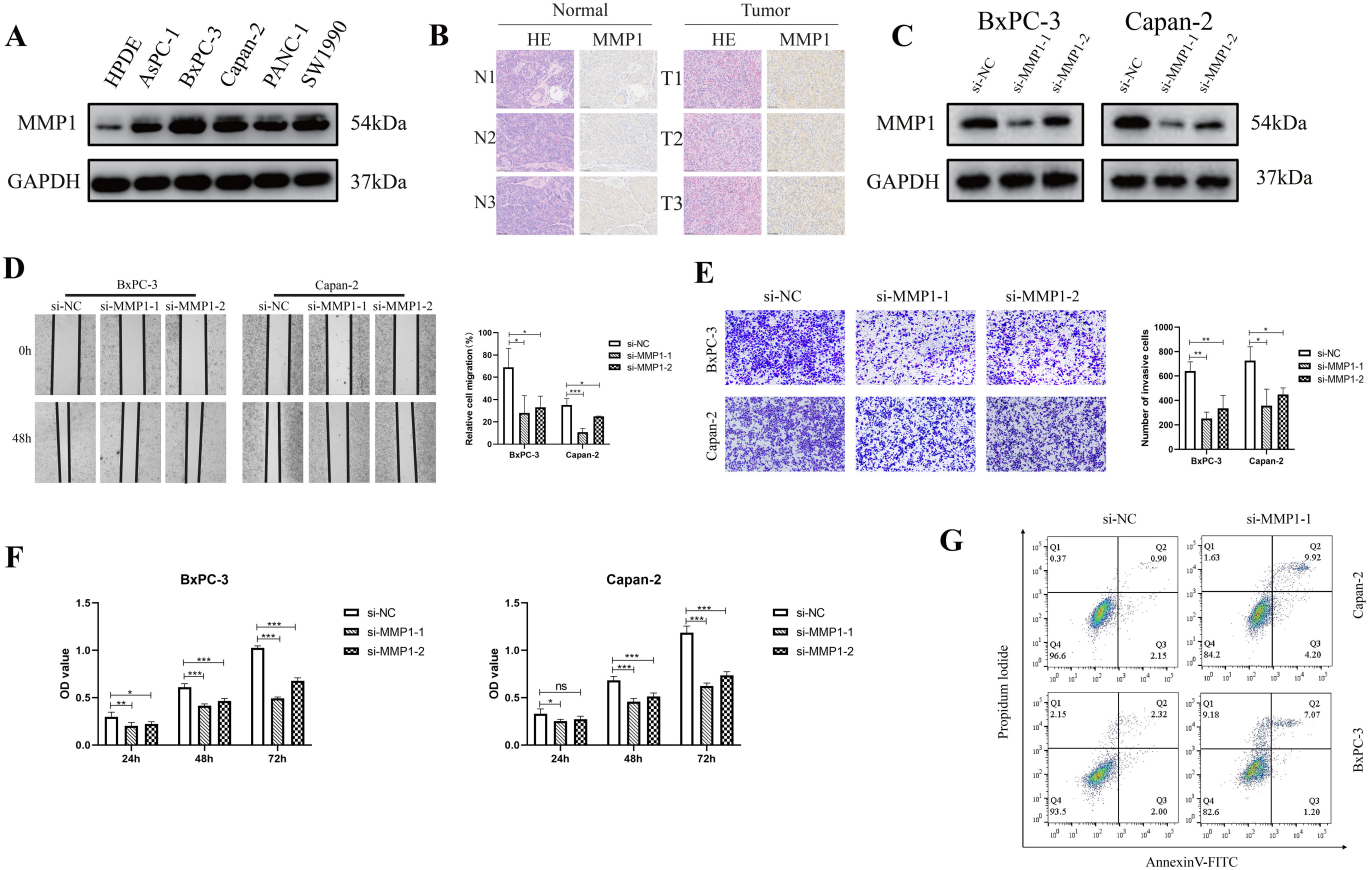
MMP1 is up-regulated in PAAD cells and is associated with proliferation, migration and invasion of PAAD cells. **(A)** The results of Western blotting confirmed the expression of MMP1 was higher in PAAD cells. **(B)** Immunohistochemistry image of MMP1 in normal pancreatic tissues and PAAD tissues. **(C)** Western blotting results showed the efficiency of siRNAs. **(D)** The results of wound-healing assays indicated the siMMP1 could restrain the migration of Capan-2 and BxPC-3 cells. **(E)** The results of transwell assays showed the ability of invasion in Capan-2 and BxPC-3 cells was decreased after transfection with siMMP1. **(F)** The proliferation of BxPC-3 and Capan-2 cells was detected by CCK8 assays. **(G)** Flow cytometry results showed that MMP1-siRNA promoted PAAD cell apoptosis. Data were presented as mean ± SD. *P < 0.05, **P < 0.01 ***P < 0.001.

A correction has been made to the section **Materials and methods**, MMP1 protein expression analysis and immunohistochemistry staining:

“MMP1 protein expression analysis and Histological analysis

Histological analysis was performed to detect histological changes by hematoxylin and eosin (HE) staining. Paraffin-embedded tissue sections (5 μm) were deparaffinized with xylene and then stained with hematoxylin and eosin. The morphology of the tissues was observed under a light microscope. In addition, sections of PC tissues and non-tumor tissues (normal controls) from patients were taken to detect MMP1 expression by immunohistochemistry. Sections were incubated at elevated temperature for 30 min for antigen retrieval, blocked with blocking solution, and incubated with anti-MMP1 antibody (10371-2-AP, Proteintech) for 1.5h at 37 °C. Sections were then incubated with IgG secondary antibody (RGAR011, Proteintech) for 30min at 37 °C. Positive MMP1 staining was observed in tissues following exposure to diaminobenzidine (DAB) and conjugation with hematoxylin.”

The original version of this article has been updated.

