# Supplementary material for: A pan-cancer analysis of the prognostic and immunological roles of matrix metalloprotease-1 (MMP1) in human tumors
Source: Front Oncol. 2023 Jan 13;12:1089550. doi: 10.3389/fonc.2022.1089550 (PMC9885257; doi:10.3389/fonc.2022.1089550)
Supplement: Supplementary file 1 [file Image_1.pdf]

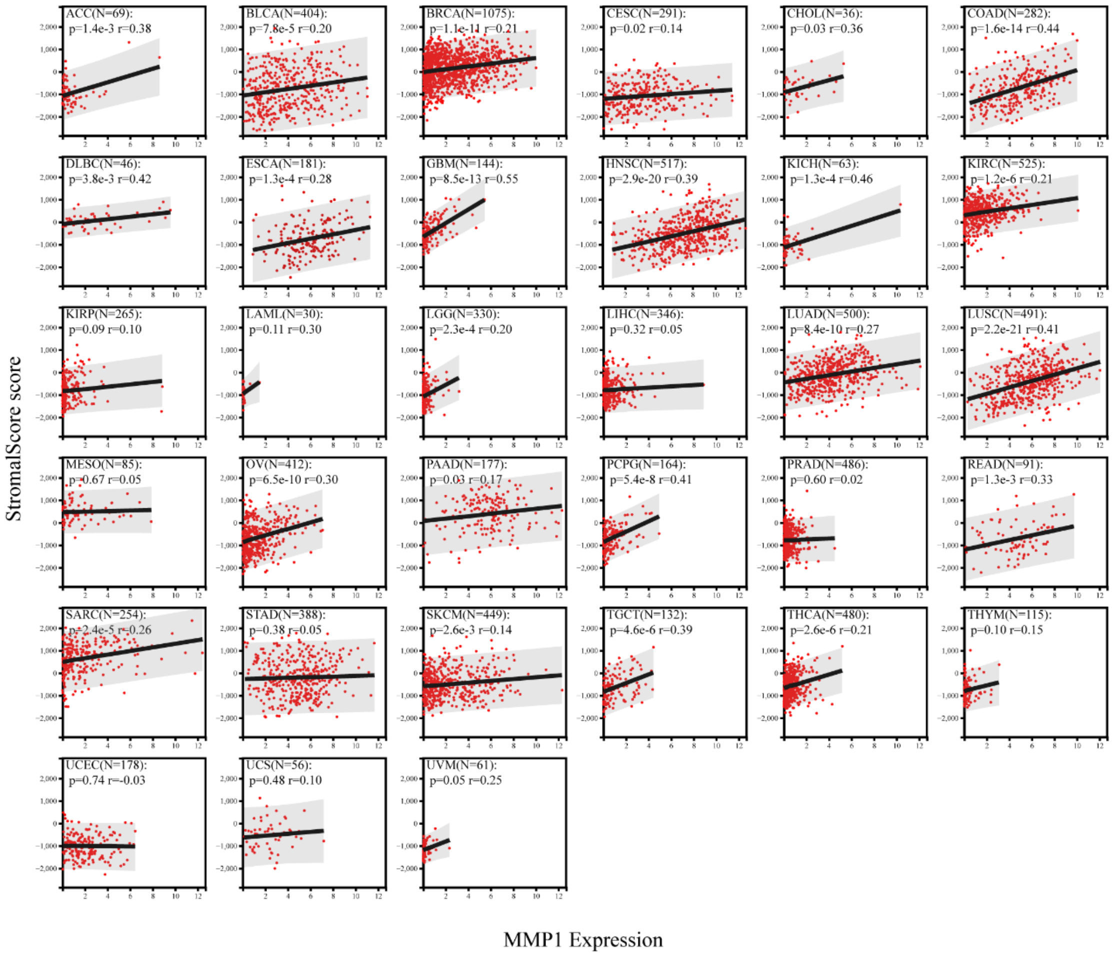

Supplementary Figure 1. Relationship between the expression of MMP1 and Stromal Score in pan-cancer. A positive correlation between MMP1 expression and stromal score in GBM, LGG, CESC, LUAD, COAD, BRCA, ESCA, SARC, HNSC, KIRC, LUSC, THCA, READ, SKCM, PAAD, OV, TGCT, PCPG, UVM, BLCA, ACC, KICH, CHOL and DLBC.

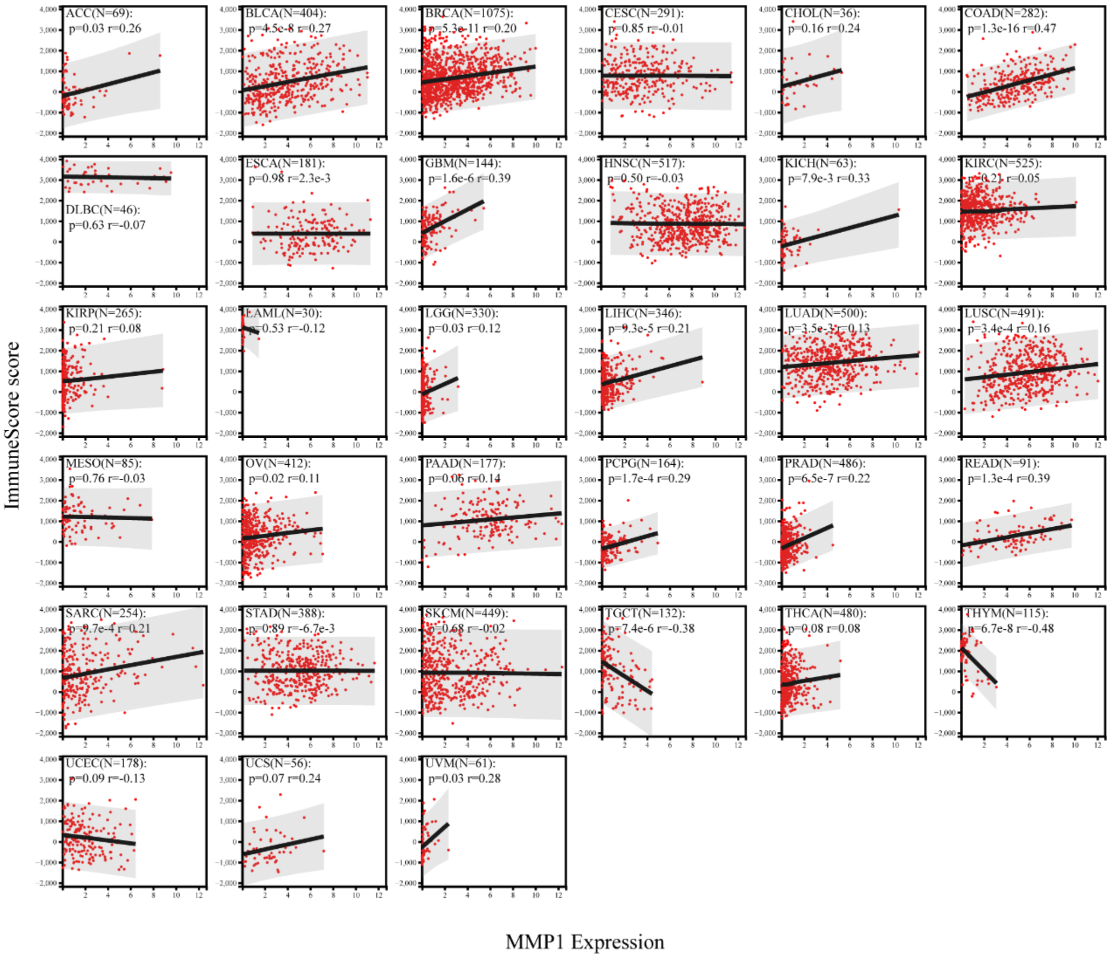

Supplementary Figure 2. Relationship between the expression of MMP1 and Immune Score in pan-cancer. We found a positive correlation between MMP1 expression and immune score in GBM, LGG, LUAD, COAD, BRCA, SARC, PRAD, LUSC, LIHC, READ, OV, PCPG, UVM, BLCA, ACC and KICH. A negative correlation was found in THYM and TGCT.

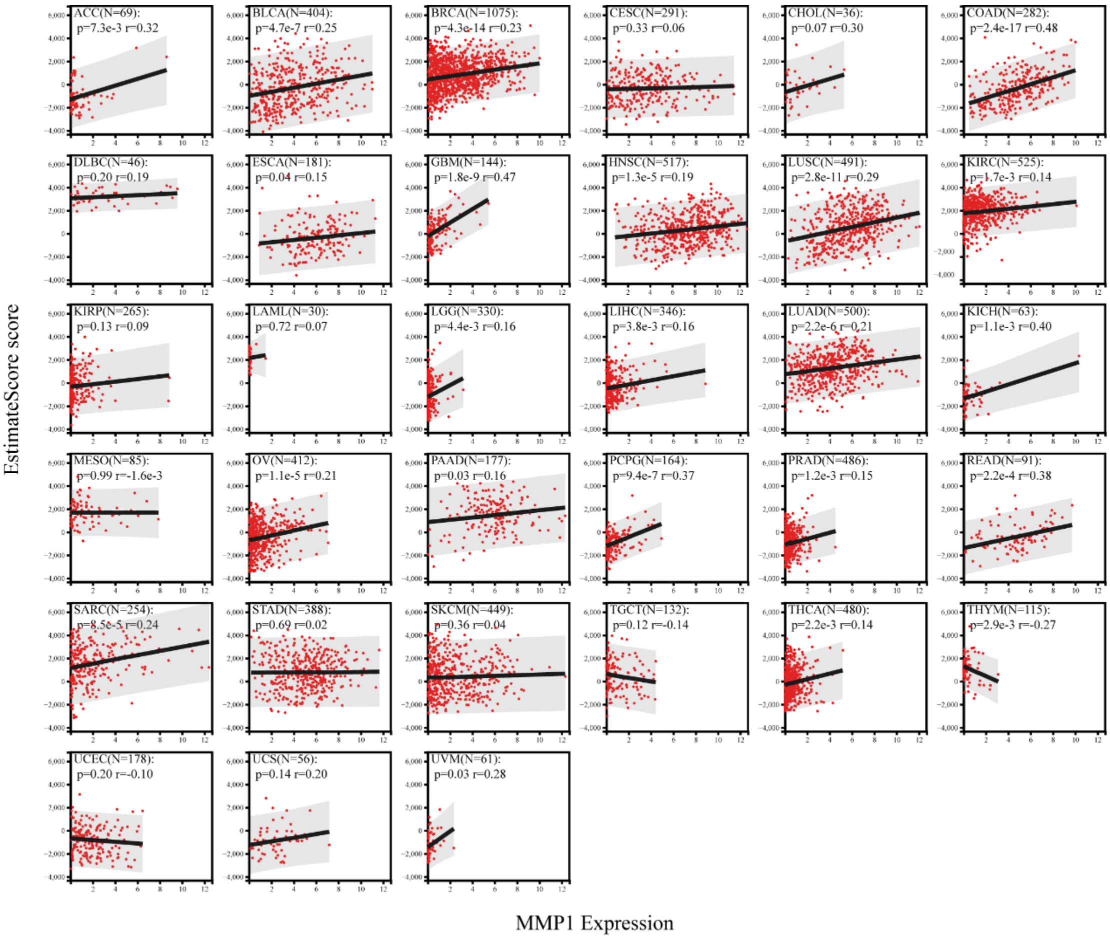

Supplementary Figure 3. Relationship between the expression of MMP1 and Estimate Score in pan-cancer. MMP1 expression was positively related to estimate score in GBM, LGG, LUAD, COAD, BRCA, ESCA, SARC, PRAD, HNSC, KIRC, LUSC, LIHC, THCA, READ, PAAD, OV, PCPG, UVM, BLCA, ACC and KICH. and a negative correlation was found in THYM. Only in THYM, a negative correlation was found.
